# Biochemical and hematological factors associated with COVID-19 severity among Gabonese patients: A retrospective cohort study

**DOI:** 10.3389/fcimb.2022.975712

**Published:** 2022-12-23

**Authors:** Nadine N’dilimabaka, Dieudonné Mounguegui Mounguegui, Sonia Etenna Lekana-Douki, Marisca Kandet Yattara, Judicaël Obame-Nkoghe, Neil Michel Longo-Pendy, Ingrid Precilya Koumba Koumba, Octavie Lauris Banga Mve-Ella, Schedy Koumba Moukouama, Cresh Emelya Dzembo, Lauriane Yacka Bolo, Prudence Biyie-Bi-Ngoghe, Guignali Laurette Mangouka, Jean-Raymond Nzenze, Jean-Bernard Lekana-Douki

**Affiliations:** ^1^ Unité Emergence des Maladies Virales, Département de Virologie, Centre Interdisciplinaire de Recherches Médicales de Franceville (CIRMF), Franceville, Gabon; ^2^ Département de Biologie, Faculté des Sciences, Université des Sciences et Techniques de Masuku (USTM), Franceville, Gabon; ^3^ Site Coronavirus, Hôpital d’Instruction des Armes d’Akanda, Libreville-Nord, Gabon; ^4^ Unité Écologie des Systèmes Vectoriels (ESV), Centre Interdisciplinaire de Recherches Médicales de Franceville (CIRMF), Franceville, Gabon; ^5^ Unité Evolution Epidémiologie et Résistances Parasitaires (UNEEREP), Centre Interdisciplinaire de Recherches Médicales de Franceville (CIRMF), Franceville, Gabon; ^6^ Département de Parasitologie-Mycologie Médecine Tropicale, Faculté de Médecine, Université des Sciences de la Sante, Libreville, Gabon

**Keywords:** Gabonese COVID-19 patients, asymptomatic, mild/moderate, severe/critic, biochemical and hematological markers, disease severity factors

## Abstract

The COVID-19 disease presents a large range of clinical manifestations and includes asymptomatic, mild, and severe cases. The level of severity is related to parameters associated with immunity, genetics, and biochemistry. Africa shows one of the lowest COVID-19 fatality rates but very few data on the biochemical markers of COVID-19 in patients and the factors associated with disease severity are available for the continent. In Gabon, the COVID-19 fatality rate is only 0.63% but almost no data on biomarkers in COVID-19 patients have been published. Both the number of COVID-19 cases and the mortality rate reported in Africa in general, and in Gabon in particular, are lower than in non-African countries. As such, understanding the factors associated with disease severity in Gabonese patients is a crucial step to better understand the disease in the African context and prepare for future COVID-19 waves and other epidemics of emerging diseases. Here, we compared biochemical and hematological markers among 753 Gabonese COVID-19 patients with asymptomatic (184/753), mild/moderate (420/753), and severe/critical (149/753) forms of the disease using an Analysis of Variance (ANOVA) or a Kruskal-Wallis (KW) test. We modeled these parameters together with comorbidities, age, and sex to predict factors associated with disease severity by using a "binomial generalized linear model" utilizing the "package" stats of R software version 4.0.2. Our results showed that almost all the biochemical and hematological parameters (except creatinine, phosphorus, D-dimers, platelets, and monocytes) varied according to disease severity. However, age and the dysfunction of organs like the kidney, liver, and lung together with the decrease of electrolytes (chloride, potassium, and sodium) are the best predictors of disease severity in Gabonese patients.

## 1 Introduction

COVID-19 (Coronavirus Disease 2019) is a pandemic pulmonary disease first reported in Wuhan in China in late December 2019 in a cluster of patients with pneumonia of unknown etiology (Lu et al., 2020; WHO, 2020). Sequencing analysis of lower respiratory samples from patients revealed that the etiological agent of the disease was a novel coronavirus later named SARS-CoV-2 (Severe Acute Respiratory Syndrome Coronavirus 2), a *Betacoronavirus* in the *Coronaviridae* family.

SARS-CoV-2 can infect all age groups but the most vulnerable are people aged more than 60 years old and those with comorbidities (e.g., cardiovascular disease, diabetes, chronic respiratory disease, cancer) (Dessie and Zewotir, 2021). The clinical presentation of COVID-19 symptoms includes mild symptoms like fever, cough, myalgia, fatigue, diarrhea, anosmia, ageusia, headache. COVID-19 also includes a wide range of life-threatening symptoms including respiratory distress syndrome, pneumonia, hepatocellular injury, hyperglycemia, dermatologic complications, encephalitis, myocarditis, liver, and kidney failure (Gupta et al., 2020; Huang et al., 2020). According to the presence and severity of these symptoms, the disease can be classified as asymptomatic, mild, moderate, severe, and critical (Who, 2020; Shen et al., 2020; Peng et al., 2020; Parasher, 2021; Kantri et al., 2021).

Many factors, such as clinical symptoms, biochemical markers, and the percentage of lung damage seen on laboratory imaging, have been found to be linked to these different levels of disease severity and can be used to predict disease outcome (Salajegheh et al., 2020; Mariam, 2022). In a meta-analysis of 42 studies including 423,117 patients, Dessie et al. found an association between comorbidities (Chronic Obstructive Pulmonary Disease, cardiovascular diseases, hypertension, obesity, cancer), acute kidney injuries, high D-dimer concentration, gender, and age with the risk of a fatal COVID-19 outcome (Dessie and Zewotir, 2021). Other factors like neutrophil activation that in turn causes tissue damage and immunothrombotic events have been found in the lungs and kidneys of COVID-19 patients (Rodrigues et al., 2020). Leukocytosis, lymphocytopenia, C-Reactive Protein (CRP), and Aspartate Aminotransferase (AST) increased with severity among COVID-19 patients (Mir et al., 2021). A reduction of platelet count is associated with high mortality and morbidity (Antoniak and Mackman, 2021). Moreover, increased levels of Lactate Dehydrogenase (LDH), Alanine Aminotransferase (ALT), and urea are indicators of disease severity and are independent risk factors for death (Chen et al., 2021). No significant difference was found in chloride concentration between severe and non-severe groups but low concentrations of sodium, potassium and calcium were inversely linked to disease severity (Chen et al., 2021).

In Africa, few data on biological characteristics of COVID-19 patients are available. In their meta-analysis of 13,568 articles, Zhu Andrew et al. (Zhu et al., 2022) found that most data came from Asia, followed by Europe, then North and South America. No data from Africa were mentioned in that study. In 2020, 439,921 articles were published on COVID-19 and referenced on Medline. Among them, only 3,767 articles reported studies on COVID-19 in Africa, representing 0.9% of all the published articles on this topic (Tonen-Wolyec et al., 2022), which highlights the lack of COVID-19 data in Africa. In Gabon, less than five articles were published in the same period of time (Tonen-Wolyec et al., 2022).

In addition, among the 514,466,108 cases of COVID-19 reported worldwide, including 6,239,016 deaths (C for SS and E (CSSE) at JHU, ) (as of May 03, 2022), only 11,446,104 cases were reported in Africa with 252,157 deaths (Gabon, 2022; Africa CDC, 2022). In Gabon, only 47,594 cases have been reported with 303 deaths (Gabon, 2022; Africa CDC, 2022). With a mortality rate of 0.63%, Gabon has one of the lowest COVID-19 fatality rates in the world. Both the relative number of COVID-19 cases per million inhabitants and the mortality rate reported in Africa, and particularly in Gabon, are lower than in non-African countries (Mariam, 2022; Osei et al., 2022; Our World in D, 2022; Worldometer, 2022). As such, understanding the factors associated with disease severity in Gabonese patients is a crucial step to better understand SARS-CoV-2 infection in the African context and prepare for future waves and other epidemics of emerging diseases.

The aim of this study was both to reduce data gaps on biochemical and hematological markers in Gabonese COVID-19 patients and to provide factors associated with disease severity.

## 2 Materials and methods

### 2.1 Data collection and patients' classification

The present study is based on retrospective data collected at the Hopital d’Instruction des Armées Akanda (HIAA) in the Estuaire province in Gabon. From March to July 2020, all suspected cases of COVID-19 patients in Gabon were systematically hospitalized or confined at home to avoid the spread of the disease in the general population. The first day of hospitalization, oropharyngeal and nasopharyngeal samples were collected for the diagnostic of COVID-19 by Polymerase Chain Reaction (PCR). Other types of analyses including clinical, biochemical, and imaging analyses were conducted in parallel. Patients with negative PCR results were discharged while those with positive results were kept at the hospital.

In this study, we collected patient information from medical records and entered them into an Excel spreadsheet. The collected information included socio-demographic information (e.g., age, sex, place of residence, occupation), clinical data (e.g., symptoms, comorbidities, oxygen saturation), biochemical markers [CRP, urea, ALT, AST, Gamma-Glutamyltransferase (GGT), Alkaline phosphatases (ALP)], electrolytes (chloride, sodium, potassium, and phosphor), hematological markers (e.g., platelets, D-dimers, hemoglobin, hematocrit, leucocytes, lymphocytes, monocytes, neutrophils, eosinophils, basophils, neutrophils), imaging tests (computed tomography), the treatments administrated and RT-PCR for SARS-CoV-2 diagnosis.

**Figure 1:**
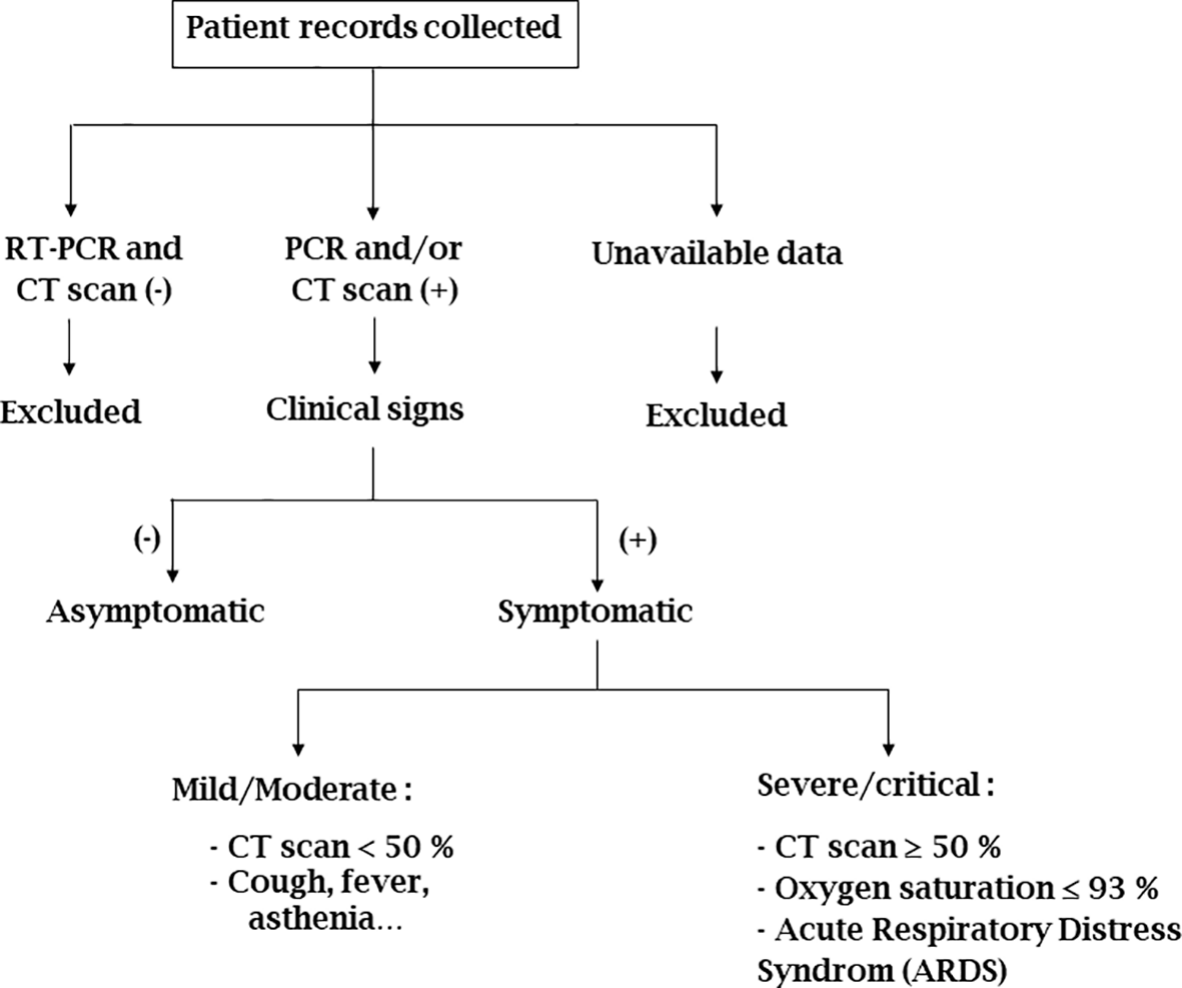
Chart on the use of data collected from COVID-19 patients for the study. (-): negative; (+): positive; CT scan < 50% and ≥ 50%: percentage of lung lesion revealed by Thoracic Computed Tomography.

Based on the clinical information, we classified patients according to three clinical statuses: asymptomatic, mild/moderate, and severe/critic. The methodology used for this classification was based on previous studies (Ranieri et al., 2012; Who, 2020; Shen et al., 2020; Peng et al., 2020; Parasher, 2021; Kantri et al., 2021) and is summarized in **Figure 1**. Patients without PCR results or those with negative PCR and CT scan results were excluded from the analysis. For remaining patients, those without any clinical signs were classified as asymptomatic while those with clinical signs were classified as mild/moderate or severe/critical, as appropriate. Patients included in the severe/critical group presented a CT scan value ≥ 50%, oxygen saturation ≤ 93% and/or Acute Respiratory Distress Syndrome (ARDS).

For this study, only biochemical and hematological markers at the time of hospitalization were used. We calculated the distribution of SARS-CoV-2 positive patients according to age groups, the mean for males and females, and the sex ratio. We calculated the mean number of COVID-19 patients according to the different levels of severity. We then compared the severity levels with organ failure markers including inflammation, kidney, and liver failure. We also compared the patients’ clinical status with electrolytes, coagulation, respiratory markers, and blood cell counts.

### 2.2 Statistical analysis

All statistical analyses were performed using *R* v*3.6.1* (https://www.r-project.org/). Numerical variables were summarized as mean ± sd (standard deviation) if they were normally distributed or otherwise as a median with IQR (interquartile range). The normality of these variables was checked using the Shapiro-Wilk test. Numerical parameters were compared between patients grouped according to COVID-19 severity level (i.e., asymptomatic, mild/moderate, and severe/critical) using an Analysis of Variance (ANOVA) or a Kruskal-Wallis (KW) test depending on the normality status of the variable. *Post-hoc* pairwise comparisons were performed accordingly, and adjusted *p-values* calculated using the Bonferroni correction. For each comparison, the test value, the degrees of freedom (df), and the *p-value* are given. All tests were two-sided, and the level of significance was set at p < 0.05.

In order to study the occurrence of the severity of COVID-19 infection according to different biochemical and immunological parameters, we used a “binomial generalized linear model” using the “package” stats (Team et al., 2018) of R software version 4.0.2 (http://cran.r-project.org). Given the possibility of significant multi-collinearity between predictor variables, a Principal Component Analysis (PCA) approach with missMDA (Josse and Husson, 2016) and FactoMineR (Lê et al., 2008) was carried out. The first two principal components obtained were used as independent variables in the model.

In this article, we compare patients assigned to the severe/critical group (severe group), patients assigned to the asymptomatic and mild/moderate groups (non-severe group), and patients assigned to the mild/moderate and severe/critical groups (symptomatic group).

## 3 Results

**Figure 2:**
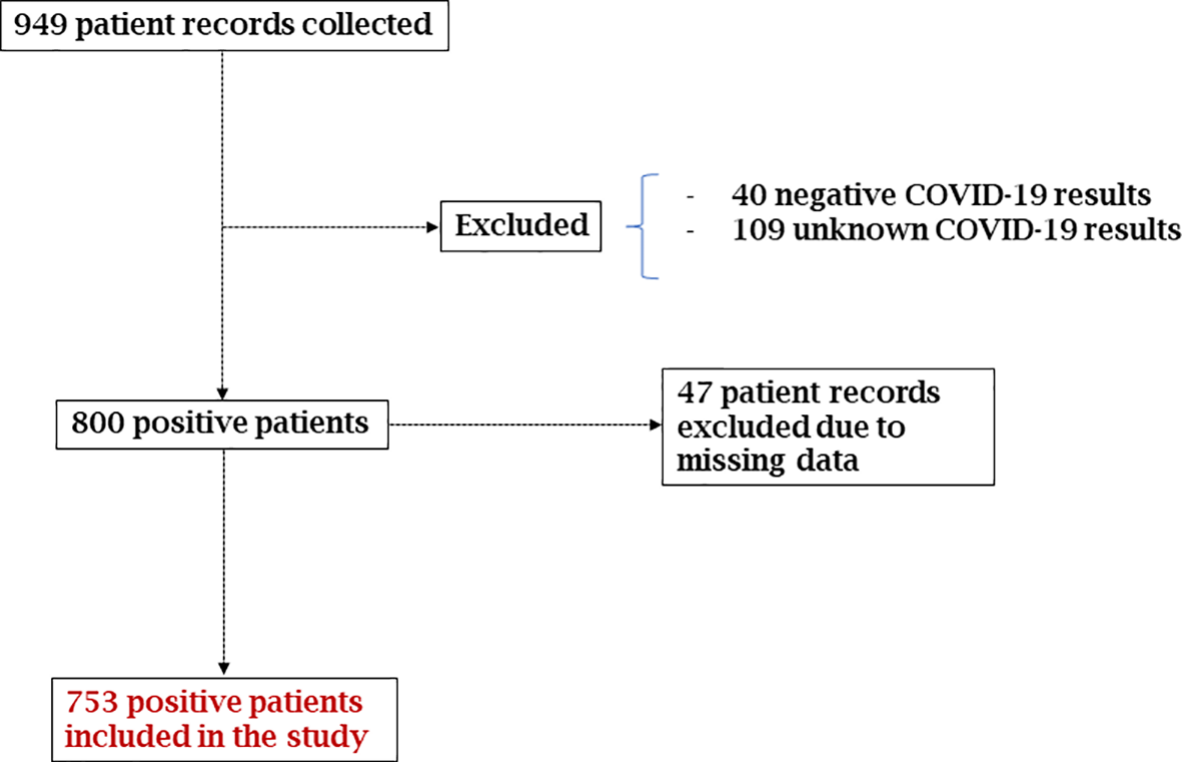
Chart of the management of COVID-19 positive patient records included in the study.

A total of 949 patient medical records were collected at the HIAA for the period between March-August 2020. One hundred and forty-nine (149) patient records were excluded due to an unknown COVID-19 status (109 patients) and negative results (40 patients). Among the remaining 800 patient medical records, 47 additional records were excluded due to missing clinical data. Ultimately, 753 patient medical records were included and analyzed in this study (**Figure 2**).

### 3.1 Demographic characteristics and clinical status of Gabonese COVID-19 patients

The median age was 40 years old (IQR 19). Men were more represented than women with a sex ratio of 1.32. The most represented age groups were adults aged 36-59 years with 54.98% (414/765) and patients aged 18-35 years old with 31.8% (240/753). On the other hand, children aged 0-17 years old with 3.32% (25/753) and people aged over 60 with 9.69% (73/753) were less represented (**Table 1**). Among the 753 patients, 56% (420/753) were assigned to the mild/moderate COVID-19 group, 24.43% (184/753) were asymptomatic, and 19.79% (149/753) were assigned to the severe/critical group (**Table 1**). The mean age increased significantly with the clinical status: 37.1 ± 12.4 years for the asymptomatic group, 41.8 ± 12.8 years for the mild/moderate group, and 47.9 ± 13 years for the severe/critical group (KW= 52.1, df = 2, p-value < 0.001) (**Table 1**). Among these patients, 5 died from COVID-19 complications during their hospitalization, leading to a mortality rate of 0.63%.

**Table 1:** COVID-19 patients’ age, sex, and comorbidities distribution rate according to the clinical status.

Characteristics		Number of			Total number (%)	KW/Chi-square	df	*p-value*
		Asymptomatic	Mild/moderate	Severe/critical				
**Age groups** **(in years)**	**[0-17]**	12	13	0	25 (3.32)	4.9 *	1	**0.02**
	**[18-35]**	77	132	31	240 (31.87)	10.6 *	2	**0.004**
	**[36-59]**	87	237	90	414 (54.98)	7.8 *	2	**0.02**
	**≥ 60**	8	37	28	73 (9.69)	1.7 *	2	0.4
	*NA	–	1	–	1(0.13)	–	–	–
	**Median**	36	40	47	40	–	–	–
	**IQR**	[29-45]	[33-51]	[38-56]	19	–	–	–
**Sex**	**Male**	123	215	90	428 (56.84)	13.9 **	2	**< 0.001**
	**Female**	61	205	58	324 (43.03)			
	*NA	–	–	1	1 (0.13)	–	–	–
	**Sex ratio** **(male to female)**	2.01	1.05	1.55	1.32	–	–	–
**Comorbidities**		44/184	159/420	81/149		36.21**	4	**< 0.0001**
**Total (%)**		184 (24.43)	420 (55.78)	149 (19.79)	753 (100)	–	–	–

*Kruskal-Wallis value; **Chi square value; Significant p-values are in bold.

Almost 38% (284/753) of the patients presented with a comorbidity. These comorbidities were associated with disease severity (X²=36.21, df=4, p<0.0001) (**Table 1**). The most common complication was high blood pressure (157/753), followed by diabetes (73/753). The less represented was Human Immunodeficiency Virus (HIV) infection (20/753) and cancer (8/753) (data not presented here).

### 3.2 Biochemical and hematological markers

**Figure 3:**
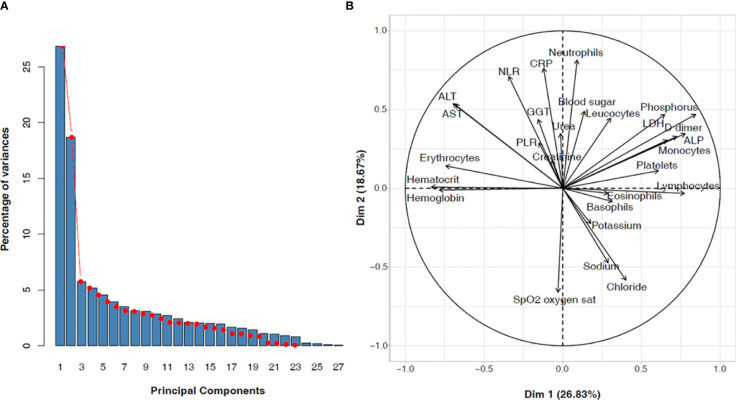
Biochemical and hematological parameters among COVID-19 Gabonese patients. **(A)** Principal components selected with the missMDA and FactoMineR approach **(B)** Weight of each variable on the principal components ALT, Alanine Aminotransferase; AST, Aspartate Aminotransferase; ALP, Alkaline phosphate; GGT, Gamma Glutamyltransferase; CRP, C-reactive protein; LDH, Lactate dehydrogenase; PLR, platelets to lymphocytes ratio; NLR, neutrophils to lymphocytes ratio.

For this study, 24 biochemical and hematological markers were analyzed for the 753 Gabonese patients. Two principal components (Dim 1 and Dim 2) were chosen by the Principal Component Analysis using the missMDA and FactoMineR packages (**Figure 3A**). These two principal components account for 45.5% of the total variance (**Figure 3B**). Dim 1 (29.5%) can be interpreted as blood disorders, characterized by leucocytes, lymphocytes, monocytes, erythrocytes, platelet count, hemoglobin, hematocrit, and D-dimers concentration (**Figure 3B**). Dim 2 (18.11%) was correlated with markers of internal organ damage (liver, kidney, lung), characterized by ALT, AST, GGT, CRP, urea, blood sugar, electrolytes (sodium, potassium, and chloride) concentrations, and oxygen saturation (**Figure 3B**).

The analysis of the 24 biochemical and hematological parameters was done according to disease severity using the Kruskall-Wallis test (**Table 2**) and additional *post-hoc* pairwise comparisons were performed accordingly (data not shown). This analysis reveals that, most of these parameters varied according to disease severity (**Table 2**).

**Table 2:** Biochemical and hematological parameters according to COVID-19 severity in Gabonese patients.

Variables	Asymptomatic	Mild/moderate	Severe/critical	KW	df	*p-value ≤*
CRP (mg/L)	3.6 ± 7.3	41.8 ± 68.2	80.7 ± 93	76.5	2	**0.001**
Urea (mmol/L)	5.12 ± 6.6	4.13 ± 2.5	5.13 ± 2.7	16.6	2	**0.001**
ALT (UI/L)	24 ± 15.3	32.14 ± 30.35	43.13 ± 35.6	17.4	2	**0.001**
AST (UI/L)	24.4 ± 7.73	34.4 ± 26.9	52.4 ± 39.1	38.2	2	**0.001**
ALP (UI/L)	47.7 ± 59	80.1 ± 55.6	86.3 ± 69.9	15.89	2	**0.001**
GGT (UI/L)	52.7 ± 36.6	65.3 ± 63.2	95.2 ± 93.4	16.24	2	**0.001**
LDH (UI/L)	231.5 ± 83.06	269.34 ± 232.4	609.16 ± 725.81	19.668	**2**	**0.0001**
Creatinine (µmol/L)	99,42 ± 20.27	112,54 ± 98.87	114.5 ± 80.87	0.04	2	0.1
Blood sugar (mmol/L)	5.44 ± 1.78	6.76 ± 4.5	8.01 ± 5.93	18.1	2	**0.0001**
Potassium (mg/dl)	3.74 ± 0.46	3.85 ± 2.83	3.62 ± 0.62	2.6	2	0.25
Sodium (mg/dl)	140 ± 3.1	138 ± 4	137 ± 7	11.4	2	**0.003**
Chlorid (mg/dl)	99.3 ± 3.9	98.3 ± 4.9	96 ± 6.7	14.5	2	**0.001**
D-dimer (µg/L)	937.865 ± 678.05	1296.249 ± 1721.8	2318.1 ± 2774	1.5	2	0.5
Platelets (x 10^3^/mm³)	214.05 ± 61.7	229.14 ± 105.74	267.51 ± 140.51	4.99	2	0.08
Erythrocytes (x 10^6^/mm³)	5.02 ± 0.71	4.67 ± 0.77	4.68 ± 0.87	13.484	2	**0.001**
Hematocrit (%)	39.9 ± 5.13	37.1 ± 5.12	36.8 ± 5.7	13.5	2	**0.001**
Hemoglobin (g/dl)	13.4 ± 1.9	12.5 ± 1.9	12.4 ± 2	11	2	**0.004**
SpO2 Oxygen saturation (%)	98.5 ± 0.91	97.8 ± 1.29	92.9 ± 6.56	140.74	2	**0.001**
Monocytes (x 10^3^/mm³)	0.6 ± 0.3	0.65 ± 0.81	0.85 ± 1.64	2.99	2	0.22
Leucocytes (x 10^3^/mm³)	7.01 ± 11.5	6.19± 4.59	7.54 ± 5.26	11.577	2	**0.003**
Lymphocytes (x 10^3^/mm³)	2.20 ± 0.668	1.90 ± 0.9	1.98 ± 2.1	20.76	2	**0.001**
Basophils (x 10^3^/mm³)	0.014 ± 0.024	0.01 ± 0.06	0.009 ± 0.025	7.22	2	**0.02**
Neutrophils (x 10^3^/mm³)	2.8 ± 1.2	3.54 ± 2.8	5 ± 3.9	21.7	2	**0.001**
Eosinophils (x 10^3^/mm³)	0.02 ± 0.05	0.009 ± 0.04	0.009 ± 0.03	8.409	2	**0.014**
PLR	103.48 ± 35.81	135.56 ± 81.35	176.96 ± 142.81	17.08	2	**0.0002**
NLR	1.35 ± 0.66	2.47 ± 3.14	3.37 ± 3.66	35.23	2	**2.24e-08**

Data are the mean ± standard error. Significant p-values are in bold. ALT, Alanine Aminotransferase; AST, Aspartate Aminotransferase; ALP, Alkaline phosphate; GGT, Gamma-GlutamylTransferase; CRP, C-reactive protein; LDH, Lactate dehydrogenase; PLR, platelets to lymphocytes ratio; NLR, neutrophils to lymphocytes ration.

#### 3.2.1 Markers of inflammation, kidney and liver failure, in patients with different COVID-19 severity levels

Increased concentrations of C-reactive protein (CRP), i.e. a plasma protein synthesized by the liver during an inflammatory process, and LDH, another inflammatory marker, were significantly associated with clinical severity: CRP (KW= 76.55, df = 2, p < 0.001) and LDH (KW= 19.67, df = 2, p < 0.0001). Similarly, the mean of urea concentration was significantly different in severe/critical cases (5.13 ± 2.7 mmol/L) compared to mild/moderate cases (4.13 ± 2.5 mmol/L) and asymptomatic cases (5.12 ± 6.6 mmol/L) (KW16.6, df = 2, p < 0.001). In contrast, creatinine concentration did not significantly change between the different groups (**Table 2**).

Liver function markers concentration also increased significantly with clinical severity: ALT (KW = 17.4, df = 2, p = 0.001), AST (KW= 38.23, df = 2, p-value = 4.99e-09), ALP (KW= 15.89, df = 2, p < 0.001), and cholangiocyte injury biomarker Gamma-GlutamylTransferase (GGT), a of biomarker located in hepatocytes and cholangiocytes, in the biliary pole (Shao et al., 2020) (KW= 16.24, df = 2, p < 0.001) (**Table 2**).

#### 3.2.2 Ionic markers

The concentration of chloride (KW= 14.497, df = 2, p < 0.001) and sodium (KW= 11.05, df = 2, p-value = 0.0039) decreased with disease severity. However, there was no significant difference in potassium concentration (KW= 2.6, df = 2, p < 0.25) (**Table 2**).

#### 3.2.3 Coagulation markers

Both D-dimer concentrations (KW = 1.5, df=2, p = 0.5) and platelet count (KW = 4.99, df=2, p = 0.08) increased with disease severity but these differences were not significant (**Table 2**).

#### 3.2.4 Respiratory and anemia markers

All parameters, erythrocytes (KW= 13.484, df = 2, p = 0.0011), hematocrit (KW= 13.484, df = 2, p < 0.001), hemoglobin (i.e., the main component of erythrocytes, responsible for the delivery of oxygen to cells and tissues and removal of carbon dioxide) (KW= 11, df = 2, p-value = 0.004), and oxygen saturation (KW= 140.74, df = 2, p < 0.001), decreased significantly according to disease severity (**Table 2**).

#### 3.2.5 White blood cells

Leucocytes (KW = 11.577, df = 2, p = 0.003) and neutrophils (KW = 21.691, df = 2, p < 0.001) increased significantly with severity while lymphocytes (KW = 20.76, df = 2, p < 0.001), eosinophils (KW = 8.4094, df = 2, p = 0.014) and basophils (KW = 7.22, df = 2, p < 0.02) decreased. There was no difference in monocyte count (KW = 2.99, df = 2, p < 0.22).

In addition, the platelets to lymphocytes ratio (PLR) (KW = 17.08, df = 2, p = 0.0002), and neutrophils to lymphocytes ratio (NLR), increased significantly according to disease severity (KW = 35.23, df = 2, p = 2.24e-08) (**Table 2**).

### 3.4 Predictors of COVID-19 severity among Gabonese patients

To determine predictors of COVID-19 severity among Gabonese patients, we considered asymptomatic and mild/moderate as the non-severe group and severe/critical as the severe group. We used the two principal components (Dim 1 and Dim 2) as independent variables in the model as well as age, sex, and different comorbidities (diabetes, cancer, high blood pressure), asthma, allergic rhinitis, pulmonary diseases, and HIV (**Table 3**). The result of our modelling analysis retained two parameters: increased age (standard error = 8.985e-03, z value = 2.981, p = 0.00287) and biochemical markers associated with Dim 2 (damage of internal organs) (standard error = 6.828e-02, z value = 7.077, p = 1.47e-12) as significant predictors to explain COVID-19 severity in Gabonese patients. These parameters were increased CRP, LDH, ALT, AST, neutrophils, NLR and decreased oxygen saturation and electrolytes (sodium, potassium, and chloride). However, comorbidities like diabetes and high blood pressure, and Dim 1 (defined by blood disorders) were not found to be independent predictors of disease severity (**Table 3**).

**Table 3:** Results of the final multivariable regression model of risk factors associated with COVID-19 severity among Gabonese patients (N=753).

Characteristics	Estimate	Std. error	z value	Pr(>|z|)
Intercept	-3.037e+00	4.322e-01	-7.028	2.10e-12 ***
Dimension 1	-3.692e-02	7.281e-02	-0.507	0.61212
Dimension 2	4.832e-01	6.828e-02	7.077	1.47e-12 ***
Sex (M)	2.835e-01	2.232e-01	1.270	0.20413
Diabetes	3.794e-01	3.361e-01	1.129	0.25896
Cancer	1.144e+00	7.630e-01	1.499	0.13390
HBP	3.775e-01	2.573e-01	1.467	0.14234
Asthma	7.808e-01	5.515e-01	1.416	0.15685
Allergic rhinitis	-1.234e-01	4.851e-01	-0.254	0.79925
Pulmonary Diseases	7.403e-01	8.001e-01	0.925	0.35486
HIV	-2.448e-01	6.361e-01	-0.385	0.70031
Age	2.679e-02	8.985e-03	2.981	0.00287 **

HBP, Hight Blood Pressure; HIV, Human Immunodeficiency Virus; **p < 0,01; ***p < 0,001.

## 4 Discussion

The median age of our study population was 40 years, and the most represented groups were adults aged 36-59 years old, followed by patients aged 18-35 years old (**Table 1**). Similar results were found both in Gabon and other countries on the African, European and American continents (Mekolo et al., 2021; Leulseged et al., 2021; Chipendo et al., 2021; Mveang Nzoghe et al., 2021). Given that the median age of the Gabonese population is 18.6 years (Varrella, 2021), we expected to find young people infected with COVID-19 (the 5-12 years and 18-24 years age groups). The fact that most infected people were in the 36-59 adult group followed by the 18-35 age group is likely because schools and universities closed when the first COVID-19 case was diagnosed in Gabon, on the 12th of March 2020, and thus only workers were exposed to the infection.

Disease severity increased with age (**Table 1**) as previously described by other authors in America, Asia and Europe (Undurraga et al., 2021; Thakur et al., 2021; Sha et al., 2021; De Pue et al., 2021; Geng et al., 2021; D’ascanio et al., 2021). This is likely due to the decrease in immunity with age (Montecino-Rodriguez et al., 2013; Mueller et al., 2020). Men were the most represented (**Table 1**) as previously shown in Zimbabwe, Cameroon and Ethiopia (Mekolo et al., 2021; Leulseged et al., 2021; Chipendo et al., 2021). This is consistent with differences in the perception of the pandemic according to gender (Galasso et al., 2020). Women are more likely to consider COVID-19 to be a public health problem than men who did not have good knowledge of barrier measures (Galasso et al., 2020). Moreover, other factors such as the innate and adaptive robust immune responses of women, the high expression of ACE2 (Angiotensin-Converting Enzyme 2) receptor by men, the expression of sex hormones (i.e., estrogen and progesterone), and the X-chromosome could also explain this difference according to gender (Jaillon et al., 2019; Salajegheh et al., 2020; Márquez et al., 2020; Shepherd et al., 2021).

In terms of clinical characteristics, the majority of patients (56%) presented a mild/moderate form of the disease (**Table 1**) consistent with previous studies in Cameroon and Gabon (Mekolo et al., 2021; Igala et al., 2021).

We also observed an association between comorbidities and severe forms of COVID-19, as previously described (Mubarik et al., 2021; Cho et al., 2021; Fang et al., 2021). Studies carried out in animal models showed a worsening of diabetes and cardiovascular diseases due to SARS-CoV-2 infection (Ma et al., 2021), suggesting that comorbidities could modify the SARS-CoV-2 inflammatory response and worsen the impact of these comorbidities.

COVID-19 mortality in both the Gabonese general population and our study population is 0.63%, lower than in many other countries (Mariam, 2022; Worldometer, 2022). To better understand this low mortality rate and the factors associated with disease severity among Gabonese COVID-19 patients, we compared the concentration of biochemical parameters and hematology in patients with different severity levels of COVID-19 in our study population. Our results showed that almost all the biochemical and hematological parameters (except creatinine, phosphorus, D-dimers, platelets, and monocytes) varied according to disease severity (**Table 2**). However, the modelling analysis of predictors associated with disease severity revealed that only increased age, elevated blood sugar levels, elevated neutrophils, NLR, markers of inflammation in kidney and liver, oxygen saturation and electrolytes are predictors of COVID-19 severity among Gabonese patients (**Table 3**).

High levels of blood glucose upon patient admission have been associated with risk factors which predict severe forms of COVID-19 and death (Sachdeva et al., 2020; Alhamar et al., 2022). Previous studies showed that SARS-CoV-2 affects the pancreas through the damage of the beta cells of the islet of Langerhans that leads to lower insulin production (Chandrashekhar and Pozzilli, 2022) and increased blood sugar levels. This hyperglycemia in turn causes Reactive Oxygen Species and increases the linkage of SARS-CoV-2 to ACE2. This linkage favorizes the cellular intrusion of the virus leading to widespread organ damage and greater disease severity (Sachdeva et al., 2020). Both hypoglycemia and direct viral interaction with the heme group of hemoglobin (the decrease of this parameter was observed during this study, see **Table 2**) lead to an increase in heme serum levels in COVID-19 patients. With harmful iron ions, both hypoglycemia and heme induces an inflammatory process (Liu and Li, 2022; Jafar et al., 2016). This inflammation results in high CRP and LDH concentrations as observed in our study. Indeed, we found that CRP and LDH concentrations increased gradually with disease severity (**Table 2**). This association was not observed in Ghana (Afriyie-Mensah et al., 2021) but other studies from different geographic locations found the same results (Kantri et al., 2021; Igala et al., 2021; Malik et al., 2021). High levels of CRP and LDH are key markers of COVID-19 progression and are associated with mortality risk factors due to disease severity (Chen et al., 2021; Gao et al., 2021; Ali et al., 2022).

High concentrations of markers of kidney and liver function (AST, ALT, GGT, ALP) according to COVID-19 severity were also observed during this study (**Table 2** and **3**), but were not found in Morocco (Kantri et al., 2021). However, similar results were found in China (Chen et al., 2020; Chen et al., 2021). High levels of urea predict mortality in UK and other countries (Aloisio et al., 2020; Zhu et al., 2022; Burke et al., 2022). Liver enzymes increase gradually with COVID-19 severity (Guan et al., 2020; Chen et al., 2021) and are high in approximately 25% of patients (Wijarnpreecha et al., 2021). This could be the result of the dysfunction of the epithelial cells of the liver and the renal system induced by SARS-CoV-2 infection (Montani et al., 2022). AST levels can result from both hepatocellular injury and muscle damage (Aloisio et al., 2020).

Increased levels of GGT are one of the most reported abnormalities in liver function and have been described in up to 51% of COVID-19 patients (Shao et al., 2020; Aloisio et al., 2020; Kumar-M et al., 2020). High levels of GGT significantly increase with disease severity and are important predictors of disease outcome. A high level of GGT may be related to oxidative stress, chronic inflammation or a marker of biliary injury (Kumar-M et al., 2020). GGT may enhance the expression of ACE2 in cholangiocytes and increase patient vulnerability to SARS-CoV-2 infection (Shao et al., 2020).

ALP levels were high in this study, as previously found in up to 58% of patients in other studies (Del et al., 2021; Benedé-Ubieto et al., 2021), and were identified as independent indicators of poor disease outcome (Aghemo et al., 2020) and hospital mortality (Del et al., 2021). ALP may reflect bone disease and systemic frailty (Aghemo et al., 2020).

In addition to the increase in liver and kidney markers, we found a decrease in sodium, and chloride ion concentrations in COVID-19 Gabonese patients as disease severity increased. However, no changes were noticed for potassium concentrations (**Table 2**). Lippi et al. and Yi Luo et al. reported a gradual decrease in potassium and sodium concentrations with COVID-19 severity (Luo et al., 2020; Lippi et al., 2020). Potassium and sodium play a significant role in the regulation of electrolyte balance. Moreover, hypokalemia and hyponatremia result in ACE2 overexpression, and increase the risk of severe forms of COVID-19 (Luo et al., 2020). In contrast, the significant decrease in chloride concentration we observed between the non-severe and severe groups was not found in other studies (Lippi et al., 2020).

We found a significant decrease in hemoglobin, hematocrit and erythrocytes depending on the severity of COVID-19 (**Table 2**). However, our modelling model did not propose these parameters as predictors of disease severity among Gabonese patients (**Table 3**). Nevertheless, the decrease of hemoglobin due to its attack by Open Reading Frame (ORF) 3 and 10, and the premature removal of less deformable erythrocytes induced by SARS-CoV-2 infection in the spleen, result in anemia, stress erythropoiesis, and hypoxia (Liu and Li, 2022; Huisjes et al., 2018; Kubánková et al., 2021; Elahi, 2022). This hypoxia may also be the result of hypoxemia induced by SARS-CoV-2 infection. The virus attacks the heme of hemoglobin which is then unable to carry oxygen and carbon dioxide, leading to respiratory distress (Liu and Li, 2022; Dhont et al., 2021). This might explain why pulse oxygen saturation gradually decreased from the asymptomatic group, to the mild/moderate group and to the severe/critical group among Gabonese patients (**Table 2**). COVID-19 patients in critical condition were associated with both a lower oxygen saturation of less than 90% and a risk of death (Mejía et al., 2020; Omran et al., 2021).

Neutrophils and leukocytes were high while lymphocytes decreased according to clinical status, as previously described in Gabon and other countries (Huang et al., 2020; Mveang Nzoghe et al., 2021; Reusch et al., 2021). SARS-CoV-2 infects lymphocytes and leads to the decrease of these cells (Huang et al., 2020). Elevated cytokine levels linked to COVID-19 severity lead to an increase of activated neutrophils that recognize the virus and coordinate its elimination with adaptive immune responses. However, in some cases like hyperglycemia, they contribute to severe forms of COVID-19 and fatal outcomes by disseminating the virus which leads to both inflammation and tissue damage (Jafar et al., 2016; Rodrigues et al., 2020). Indeed, neutrophils intensively infiltrate the lung and induce an inflammatory process (Parthasarathi et al., 2022). In a metanalysis of 16,205 patients, Parthasarathi et al. found an association between NLR on admission and a risk factor of both disease severity and mortality (Parthasarathi et al., 2022).

The variation of biomarkers according to disease severity found in this study are in accordance with the literature from non-African COVID-19 patients suggesting that other factors may be responsible for the difference in mortality observed. Previous immunity acquired from infection with animal coronaviruses, BCG vaccination to *Mycobacterium bovis* (Bacillus Calmette–Guerin, a live attenuated vaccine for tuberculosis), and malaria infection, together with genetic factors and weather conditions, have been hypothesized as factors that limit the impact of COVID-19 in Africa (Mariam, 2022).

### 4.1 Study limitations

Our study presents several limitations. Data analysis was done retrospectively and concerned only a single healthcare center. The low number of patients deceased (only five) in our cohort study did not allow us to correlate parameters and the outcome of the disease. Many data like electrolytes (calcium and magnesium), coagulation (fibrinogen) and inflammatory markers (cytokines) are also missing. We were therefore unable to complete the analysis and get a more precise model of COVID-19 severity and mortality among Gabonese patients at this time. Further studies with more patients and additional parameters are required to validate these results.

## 5 Conclusion

We found that almost all the hematological factors and biomarkers varied according to disease severity among Gabonese COVID-19 patients. However, our model found that increased age, inflammatory markers, neutrophils, NLR, the dysfunction of internal organs (liver, kidney and lungs), together with the decrease of electrolytes like chloride and sodium, are the best factors associated with disease severity in the Gabonese context. Our data are in accordance with previous studies from the literature in other continents. Our findings suggest that other factors may explain the difference in COVID-19 mortality between Gabon and other continents. Further investigations on immunity, genetic and meteorological factors are needed to better explain these differences.

## Data availability statement

The raw data supporting the conclusions of this article will be made available by the authors, without undue reservation.

## Ethics statement

This study was approved by the Comité National d’Ethique pour la Recherche (CNER, Gabonese National Ethics Committee for Research) and registered under the number N°0003/2020/CNER/SG/P.

## Author contributions

NN, SELD, and JBLD contributed to the design of the study. DMM, MKY, PBBN, GLM, and JRN cared for the COVID-19 patients and provided the patients' medical records. NN, OLBME, CED, and LYB collected the patients' data from the medical records. IPKK, SKM, OLBME, CEN, and LYB entered the patients' data in an Excel file. JON and NMLP performed the statistical analysis. NN, SELD, and IPKK analyzed the data. NN prepared the first draft of the manuscript, and edited. NN and JBLD reviewed the manuscript and supervised the work. All authors contributed to the article and approved the submitted version of the manuscript.
